# 2-Amino-*N*-[3-(2-chloro­benzo­yl)-5-ethyl­thio­phen-2-yl]acetamide

**DOI:** 10.1107/S1600536812003261

**Published:** 2012-01-31

**Authors:** Hoong-Kun Fun, Suchada Chantrapromma, A. S. Dayananda, H. S. Yathirajan, A. R. Ramesha

**Affiliations:** a X-ray Crystallography Unit, School of Physics, Universiti Sains Malaysia, 11800 USM, Penang, Malaysia; b Crystal Materials Research Unit, Department of Chemistry, Faculty of Science, Prince of Songkla University, Hat-Yai, Songkhla 90112, Thailand; c Department of Studies in Chemistry, University of Mysore, Manasagangotri, Mysore 570 006, India; d R. L. Fine Chem, Bangalore 560 064, India

## Abstract

In the title compound, C_15_H_15_ClN_2_O_2_S, the 2-amino­acetamide N—C(=O)—C—N unit is approximately planar, with an r.m.s. deviation of 0.020 (4) Å. The central thio­phene ring makes dihedral angles of 7.84 (11) and 88.11 (11)°, respectively, with the 2-amino­acetamide unit and the 2-chloro­phenyl ring. An intra­molecular N—H⋯O hydrogen bond generates an *S*(6) ring motif. In the crystal, mol­ecules are linked by an N—H⋯O hydrogen bond and weak C—H⋯O inter­actions into a chain along the *c* axis. A C—H⋯π inter­action is also present.

## Related literature

For bond-length data, see: Allen *et al.* (1987 ▶). For related literature on hydrogen-bond motifs, see: Bernstein *et al.* (1995 ▶). For background to and activities of etizolam and thio­phene derivatives, see: Gewald & Schindler (1990 ▶); Jagadees Babu *et al.* (2011 ▶); Shafeeque *et al.* (1999 ▶); Nakamura & Mukasa (1992 ▶); Nakanishi *et al.* (1973 ▶); Ramanathan & Namboothiri (1978 ▶). For related structures, see: Dockendorff *et al.* (2006 ▶); Ferreira de Lima *et al.* (2009 ▶); Nogueira *et al.* (2010 ▶). For the stability of the temperature controller, see: Cosier & Glazer (1986 ▶).
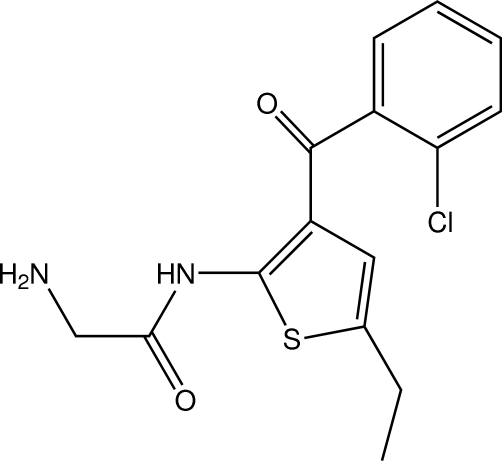


## Experimental

### 

#### Crystal data

C_15_H_15_ClN_2_O_2_S
*M* *_r_* = 322.80
Monoclinic, 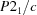

*a* = 13.9784 (11) Å
*b* = 13.5565 (11) Å
*c* = 8.3334 (7) Å
β = 91.233 (1)°
*V* = 1578.8 (2) Å^3^
*Z* = 4
Mo *K*α radiation
μ = 0.38 mm^−1^
*T* = 293 K
0.56 × 0.41 × 0.28 mm

#### Data collection

Bruker APEX DUO CCD area-detector diffractometer
Absorption correction: multi-scan (*SADABS*; Bruker, 2009 ▶) *T*_min_ = 0.817, *T*_max_ = 0.902
16034 measured reflections
4184 independent reflections
3180 reflections with *I* > 2σ(*I*)
*R*_int_ = 0.020

#### Refinement

*R*[*F* ^2^ > 2σ(*F* ^2^)] = 0.049
*wR*(*F* ^2^) = 0.163
*S* = 1.05
4184 reflections
191 parameters
H-atom parameters constrained
Δρ_max_ = 0.47 e Å^−3^
Δρ_min_ = −0.28 e Å^−3^

### 

Data collection: *APEX2* (Bruker, 2009 ▶); cell refinement: *SAINT* (Bruker, 2009 ▶); data reduction: *SAINT*; program(s) used to solve structure: *SHELXTL* (Sheldrick, 2008 ▶); program(s) used to refine structure: *SHELXTL*; molecular graphics: *SHELXTL*; software used to prepare material for publication: *SHELXTL* and *PLATON* (Spek, 2009 ▶).

## Supplementary Material

Crystal structure: contains datablock(s) global, I. DOI: 10.1107/S1600536812003261/is5060sup1.cif

Structure factors: contains datablock(s) I. DOI: 10.1107/S1600536812003261/is5060Isup2.hkl

Supplementary material file. DOI: 10.1107/S1600536812003261/is5060Isup3.cml

Additional supplementary materials:  crystallographic information; 3D view; checkCIF report

## Figures and Tables

**Table 1:** Hydrogen-bond geometry (Å, °) *Cg*1 is the centroid of the thio­phene C8/C9/S1/C10/C11 ring.

*D*—H⋯*A*	*D*—H	H⋯*A*	*D*⋯*A*	*D*—H⋯*A*
N1—H1*N*1⋯O1	0.83	2.15	2.771 (2)	132
N2—H2*N*2⋯O2^i^	0.87	2.48	3.118 (3)	131
C15—H15*B*⋯O2^i^	0.97	2.37	3.120 (3)	134
C15—H15*A*⋯*Cg*1^ii^	0.97	2.75	3.530 (2)	238

Symmetry codes: (i) 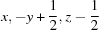; (ii) 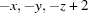.

**Fig. 1.**
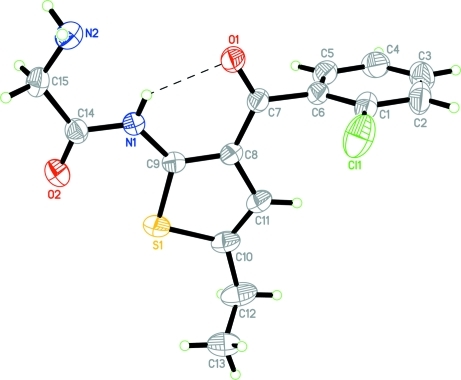
The molecular structure of the title compound, showing 40% probability displacement ellipsoids and the atom-numbering scheme. The intramolecular N—H···O hydrogen bond is shown as a dash line.

**Fig. 2.**
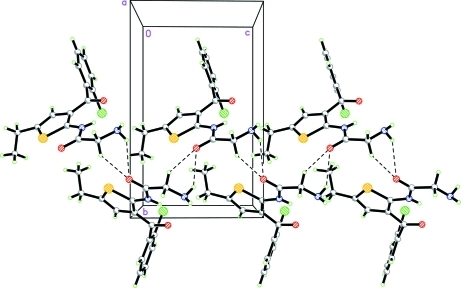
The crystal packing of the title compound viewed along the a axis, showing a chain running along the c axis. Hydrogen bonds are shown as dashed lines.

## supplementary crystallographic information

### Comment

The title compound is an intermediate in the synthesis of a drug known as "etizolam" which possesses potent hypnotic properties (Nakamura & Mukasa, 1992). Thiophenes and their biheterocycles have received considerable attention during last two decades as they are endowed with variety of biological activities and have wide range of therapeutic properties (Gewald & Schindler, 1990; Ramanathan & Namboothiri, 1978). Thiophene derivatives possess different pharmacological and biological properties, of which the more potent properties are the anticonvulsant, anti-inflammatory and antibacterial activities (Jagadees Babu *et al.*, 2011; Shafeeque *et al.*, 1999). In view of the importance of thiophenes, the crystal structure of the title compound (I) is reported.

In the molecule of (I), C_15_H_15_ClN_2_O_2_S, the central thiophene ring makes a dihedral angle of 88.11 (11)° with the 2-chlorophenyl ring. The 2-aminoacetamide moiety is co-planar with the thiophene ring with an r.m.s. deviation of 0.067 (2) Å for the ten non-H atoms (C8–C11/C14-C15, S1, O2 N1 and N2) (Fig. 1) and with torsion angles C9–N1–C14–O2 = -1.6 (3)°, C9–N1–C14–C15 = 178.49 (18)° and N1–C14–C15–N2 = -6.9 (3)°. The orientation of the ethyl group with respect to the thiophene ring can be reflected by the torsion angle C11–C10–C12–C13 = 118.6 (4)° which indicates the (+)-anti-clinal conformation. An intramolecular N1—H1N1···O1 hydrogen bond generates an S(6) ring motif (Bernstein *et al.*, 1995). Bond distances of (I) are in normal range (Allen *et al.*, 1987) and comparable with the related structures (Dockendorff *et al.*, 2006; Ferreira de Lima *et al.*, 2009; Nogueira *et al.*, 2010).

In the crystal packing (Fig. 2), the molecules are linked by intermolecular N—H···O(acetamide) hydrogen bonds and weak C—H···O(acetamide) interactions (Table 1) into chains along the *c* axis. Weak C—H···π interactions are present (Table1).

### Experimental

The title compound was synthesized by the literature method (Nakanishi *et al.*, 1973). Yellow block-shaped single crystals of the title compound suitable for *X*-ray structure determination were recrystalized from C_2_H_5_OH/DMSO (1:1 *v*/*v*) by slow evaporation of the solvent at room temperature after several days (m.p. 417–419 K).

### Refinement

All H atoms were positioned geometrically and allowed to ride on their parent atoms, with d(N—H) = 0.83 Å for NH, 0.87 Å for NH_2_, and d(C—H) = 0.93 Å for aromatic, 0.97 Å for CH_2_ and 0.96 Å for CH_3_ groups. The *U*_iso_(H) values were constrained to be 1.5*U*_eq_ of the carrier atom for methyl H atoms and 1.2*U*_eq_ for the remaining H atoms. A rotating group model was used for the methyl groups.

### Crystal data

**Table:**

C_15_H_15_ClN_2_O_2_S	*F*(000) = 672
*M**_r_* = 322.80	*D*_x_ = 1.358 Mg m^−^^3^
Monoclinic, *P*2_1_/*c*	Melting point = 417–419 K
Hall symbol: -P 2ybc	Mo *K*α radiation, λ = 0.71073 Å
*a* = 13.9784 (11) Å	Cell parameters from 4184 reflections
*b* = 13.5565 (11) Å	θ = 1.5–29.0°
*c* = 8.3334 (7) Å	µ = 0.38 mm^−^^1^
β = 91.233 (1)°	*T* = 293 K
*V* = 1578.8 (2) Å^3^	Block, yellow
*Z* = 4	0.56 × 0.41 × 0.28 mm

### Data collection

**Table:**

Bruker APEX DUO CCD area-detector diffractometer	4184 independent reflections
Radiation source: sealed tube	3180 reflections with *I* > 2σ(*I*)
graphite	*R*_int_ = 0.020
φ and ω scans	θ_max_ = 29.0°, θ_min_ = 1.5°
Absorption correction: multi-scan (*SADABS*; Bruker, 2009)	*h* = −18→19
*T*_min_ = 0.817, *T*_max_ = 0.902	*k* = −18→17
16034 measured reflections	*l* = −11→11

### Refinement

**Table:**

Refinement on *F*^2^	Primary atom site location: structure-invariant direct methods
Least-squares matrix: full	Secondary atom site location: difference Fourier map
*R*[*F*^2^ > 2σ(*F*^2^)] = 0.049	Hydrogen site location: inferred from neighbouring sites
*wR*(*F*^2^) = 0.163	H-atom parameters constrained
*S* = 1.05	*w* = 1/[σ^2^(*F*_o_^2^) + (0.079*P*)^2^ + 0.4642*P*] where *P* = (*F*_o_^2^ + 2*F*_c_^2^)/3
4184 reflections	(Δ/σ)_max_ = 0.001
191 parameters	Δρ_max_ = 0.47 e Å^−^^3^
0 restraints	Δρ_min_ = −0.28 e Å^−^^3^

### Special details

**Table:**

Experimental. The crystal was placed in the cold stream of an Oxford Cryosystems Cobra open-flow nitrogen cryostat (Cosier & Glazer, 1986) operating at 100.0 (1) K.
Geometry. All esds (except the esd in the dihedral angle between two l.s. planes) are estimated using the full covariance matrix. The cell esds are taken into account individually in the estimation of esds in distances, angles and torsion angles; correlations between esds in cell parameters are only used when they are defined by crystal symmetry. An approximate (isotropic) treatment of cell esds is used for estimating esds involving l.s. planes.
Refinement. Refinement of F^2^ against ALL reflections. The weighted R-factor wR and goodness of fit S are based on F^2^, conventional R-factors R are based on F, with F set to zero for negative F^2^. The threshold expression of F^2^ > 2sigma(F^2^) is used only for calculating R-factors(gt) etc. and is not relevant to the choice of reflections for refinement. R-factors based on F^2^ are statistically about twice as large as those based on F, and R- factors based on ALL data will be even larger.

### Fractional atomic coordinates and isotropic or equivalent isotropic displacement parameters (Å^2^)

**Table:**

	*x*	*y*	*z*	*U*_iso_*/*U*_eq_	
Cl1	0.44203 (5)	0.00271 (7)	0.78182 (12)	0.1059 (3)	
S1	0.12494 (4)	0.13080 (4)	1.17920 (6)	0.06549 (19)	
O1	0.19142 (11)	−0.04825 (14)	0.72869 (17)	0.0736 (5)	
O2	−0.03578 (13)	0.17865 (14)	1.0035 (2)	0.0834 (5)	
N1	0.06864 (11)	0.08376 (12)	0.87282 (19)	0.0516 (4)	
H1N1	0.0845	0.0597	0.7864	0.062*	
N2	−0.02762 (16)	0.09813 (17)	0.5929 (3)	0.0771 (5)	
H1N2	−0.0804	0.0735	0.5496	0.093*	
H2N2	−0.0066	0.1416	0.5262	0.093*	
C1	0.40677 (15)	−0.11245 (19)	0.8517 (3)	0.0658 (5)	
C2	0.47451 (19)	−0.1860 (3)	0.8745 (3)	0.0863 (8)	
H2A	0.5387	−0.1741	0.8543	0.104*	
C3	0.4451 (2)	−0.2771 (2)	0.9274 (3)	0.0897 (8)	
H3A	0.4899	−0.3274	0.9402	0.108*	
C4	0.3506 (2)	−0.2951 (2)	0.9617 (3)	0.0834 (7)	
H4A	0.3319	−0.3566	0.9988	0.100*	
C5	0.28426 (17)	−0.22083 (18)	0.9404 (3)	0.0694 (6)	
H5A	0.2206	−0.2324	0.9645	0.083*	
C6	0.31130 (13)	−0.12879 (15)	0.8834 (2)	0.0545 (4)	
C7	0.23554 (13)	−0.05129 (16)	0.8577 (2)	0.0540 (4)	
C8	0.21449 (13)	0.01234 (14)	0.9924 (2)	0.0506 (4)	
C9	0.13424 (13)	0.07179 (14)	0.9968 (2)	0.0497 (4)	
C10	0.23183 (18)	0.07790 (18)	1.2498 (3)	0.0677 (6)	
C11	0.26932 (16)	0.01788 (16)	1.1402 (2)	0.0606 (5)	
H11A	0.3258	−0.0171	1.1580	0.073*	
C12	0.2683 (3)	0.1027 (3)	1.4170 (3)	0.1028 (10)	
H12A	0.2203	0.0829	1.4930	0.123*	
H12B	0.3253	0.0639	1.4394	0.123*	
C13	0.2909 (3)	0.2061 (3)	1.4438 (5)	0.1304 (15)	
H13A	0.3160	0.2147	1.5510	0.196*	
H13B	0.2339	0.2450	1.4296	0.196*	
H13C	0.3378	0.2270	1.3684	0.196*	
C14	−0.01269 (14)	0.13727 (14)	0.8807 (3)	0.0568 (4)	
C15	−0.07268 (15)	0.14234 (17)	0.7292 (3)	0.0642 (5)	
H15A	−0.1331	0.1092	0.7468	0.077*	
H15B	−0.0865	0.2109	0.7050	0.077*	

### Atomic displacement parameters (Å^2^)

**Table:**

	*U*^11^	*U*^22^	*U*^33^	*U*^12^	*U*^13^	*U*^23^
Cl1	0.0672 (4)	0.1184 (7)	0.1321 (7)	−0.0118 (4)	0.0012 (4)	0.0488 (5)
S1	0.0777 (4)	0.0644 (3)	0.0544 (3)	−0.0016 (2)	0.0008 (2)	−0.0138 (2)
O1	0.0712 (9)	0.1004 (12)	0.0487 (7)	0.0291 (9)	−0.0113 (6)	−0.0111 (7)
O2	0.0772 (11)	0.0864 (12)	0.0864 (11)	0.0238 (9)	0.0016 (9)	−0.0235 (10)
N1	0.0509 (8)	0.0529 (9)	0.0509 (8)	0.0057 (7)	−0.0018 (6)	−0.0039 (6)
N2	0.0811 (13)	0.0784 (13)	0.0710 (12)	0.0116 (11)	−0.0191 (10)	−0.0050 (10)
C1	0.0517 (10)	0.0825 (15)	0.0631 (12)	0.0085 (10)	−0.0008 (9)	0.0073 (11)
C2	0.0599 (13)	0.119 (2)	0.0801 (16)	0.0295 (14)	−0.0003 (11)	0.0058 (16)
C3	0.095 (2)	0.0887 (19)	0.0849 (17)	0.0402 (16)	−0.0130 (14)	−0.0049 (15)
C4	0.099 (2)	0.0647 (14)	0.0851 (17)	0.0086 (13)	−0.0192 (14)	−0.0007 (12)
C5	0.0655 (13)	0.0703 (14)	0.0720 (13)	−0.0003 (10)	−0.0114 (10)	0.0000 (11)
C6	0.0497 (9)	0.0654 (12)	0.0483 (9)	0.0070 (8)	−0.0045 (7)	−0.0031 (8)
C7	0.0470 (9)	0.0654 (11)	0.0494 (9)	0.0045 (8)	−0.0027 (7)	0.0002 (8)
C8	0.0477 (9)	0.0560 (10)	0.0478 (9)	−0.0027 (7)	−0.0047 (7)	−0.0009 (7)
C9	0.0518 (9)	0.0492 (9)	0.0480 (9)	−0.0050 (7)	−0.0003 (7)	−0.0030 (7)
C10	0.0822 (14)	0.0698 (13)	0.0505 (10)	−0.0120 (11)	−0.0132 (10)	−0.0024 (9)
C11	0.0608 (11)	0.0649 (12)	0.0555 (10)	−0.0065 (9)	−0.0135 (9)	0.0017 (9)
C12	0.129 (3)	0.121 (2)	0.0574 (13)	−0.024 (2)	−0.0235 (15)	−0.0127 (15)
C13	0.131 (3)	0.144 (3)	0.114 (3)	0.016 (2)	−0.040 (2)	−0.072 (3)
C14	0.0534 (10)	0.0485 (10)	0.0686 (12)	0.0021 (8)	0.0015 (9)	−0.0005 (9)
C15	0.0538 (11)	0.0601 (12)	0.0784 (14)	0.0042 (9)	−0.0054 (9)	0.0132 (10)

### Geometric parameters (Å, °)

**Table:**

Cl1—C1	1.741 (3)	C5—C6	1.390 (3)
S1—C9	1.7248 (18)	C5—H5A	0.9300
S1—C10	1.748 (3)	C6—C7	1.504 (3)
O1—C7	1.229 (2)	C7—C8	1.451 (3)
O2—C14	1.217 (3)	C8—C9	1.382 (3)
N1—C14	1.351 (2)	C8—C11	1.438 (3)
N1—C9	1.376 (2)	C10—C11	1.338 (3)
N1—H1N1	0.8253	C10—C12	1.511 (3)
N2—C15	1.441 (3)	C11—H11A	0.9300
N2—H1N2	0.8805	C12—C13	1.454 (5)
N2—H2N2	0.8655	C12—H12A	0.9700
C1—C6	1.384 (3)	C12—H12B	0.9700
C1—C2	1.386 (3)	C13—H13A	0.9600
C2—C3	1.376 (5)	C13—H13B	0.9600
C2—H2A	0.9300	C13—H13C	0.9600
C3—C4	1.379 (4)	C14—C15	1.502 (3)
C3—H3A	0.9300	C15—H15A	0.9700
C4—C5	1.378 (3)	C15—H15B	0.9700
C4—H4A	0.9300		
			
C9—S1—C10	91.49 (10)	N1—C9—C8	125.22 (16)
C14—N1—C9	125.06 (17)	N1—C9—S1	123.04 (14)
C14—N1—H1N1	119.8	C8—C9—S1	111.74 (13)
C9—N1—H1N1	114.9	C11—C10—C12	129.4 (3)
C15—N2—H1N2	96.0	C11—C10—S1	111.37 (15)
C15—N2—H2N2	112.5	C12—C10—S1	119.2 (2)
H1N2—N2—H2N2	106.7	C10—C11—C8	114.0 (2)
C6—C1—C2	121.2 (2)	C10—C11—H11A	123.0
C6—C1—Cl1	119.25 (17)	C8—C11—H11A	123.0
C2—C1—Cl1	119.6 (2)	C13—C12—C10	115.1 (3)
C3—C2—C1	118.8 (3)	C13—C12—H12A	108.5
C3—C2—H2A	120.6	C10—C12—H12A	108.5
C1—C2—H2A	120.6	C13—C12—H12B	108.5
C2—C3—C4	121.2 (2)	C10—C12—H12B	108.5
C2—C3—H3A	119.4	H12A—C12—H12B	107.5
C4—C3—H3A	119.4	C12—C13—H13A	109.5
C5—C4—C3	119.3 (3)	C12—C13—H13B	109.5
C5—C4—H4A	120.3	H13A—C13—H13B	109.5
C3—C4—H4A	120.3	C12—C13—H13C	109.5
C4—C5—C6	120.8 (2)	H13A—C13—H13C	109.5
C4—C5—H5A	119.6	H13B—C13—H13C	109.5
C6—C5—H5A	119.6	O2—C14—N1	121.8 (2)
C1—C6—C5	118.6 (2)	O2—C14—C15	122.17 (19)
C1—C6—C7	122.7 (2)	N1—C14—C15	116.03 (18)
C5—C6—C7	118.68 (18)	N2—C15—C14	113.44 (17)
O1—C7—C8	123.42 (18)	N2—C15—H15A	108.9
O1—C7—C6	119.14 (18)	C14—C15—H15A	108.9
C8—C7—C6	117.31 (16)	N2—C15—H15B	108.9
C9—C8—C11	111.39 (17)	C14—C15—H15B	108.9
C9—C8—C7	123.05 (16)	H15A—C15—H15B	107.7
C11—C8—C7	125.47 (18)		
			
C6—C1—C2—C3	1.0 (4)	C14—N1—C9—S1	−4.1 (3)
Cl1—C1—C2—C3	−179.0 (2)	C11—C8—C9—N1	178.71 (18)
C1—C2—C3—C4	−1.7 (4)	C7—C8—C9—N1	−4.8 (3)
C2—C3—C4—C5	0.9 (4)	C11—C8—C9—S1	−1.3 (2)
C3—C4—C5—C6	0.7 (4)	C7—C8—C9—S1	175.21 (15)
C2—C1—C6—C5	0.6 (3)	C10—S1—C9—N1	−178.53 (17)
Cl1—C1—C6—C5	−179.48 (17)	C10—S1—C9—C8	1.49 (16)
C2—C1—C6—C7	−179.0 (2)	C9—S1—C10—C11	−1.31 (19)
Cl1—C1—C6—C7	1.0 (3)	C9—S1—C10—C12	179.3 (2)
C4—C5—C6—C1	−1.4 (3)	C12—C10—C11—C8	−179.9 (3)
C4—C5—C6—C7	178.2 (2)	S1—C10—C11—C8	0.8 (3)
C1—C6—C7—O1	92.5 (3)	C9—C8—C11—C10	0.3 (3)
C5—C6—C7—O1	−87.0 (3)	C7—C8—C11—C10	−176.1 (2)
C1—C6—C7—C8	−91.3 (2)	C11—C10—C12—C13	118.6 (4)
C5—C6—C7—C8	89.1 (2)	S1—C10—C12—C13	−62.2 (4)
O1—C7—C8—C9	10.0 (3)	C9—N1—C14—O2	−1.6 (3)
C6—C7—C8—C9	−166.00 (18)	C9—N1—C14—C15	178.49 (18)
O1—C7—C8—C11	−174.0 (2)	O2—C14—C15—N2	173.2 (2)
C6—C7—C8—C11	10.0 (3)	N1—C14—C15—N2	−6.9 (3)
C14—N1—C9—C8	175.84 (19)		

### Hydrogen-bond geometry (Å, °)

**Table:**

*Cg*1 is the centroid of the thiophene C8/C9/S1/C10/C11 ring.

**Table:**

*D*—H···*A*	*D*—H	H···*A*	*D*···*A*	*D*—H···*A*
N1—H1N1···O1	0.83	2.15	2.771 (2)	132
N2—H2N2···O2^i^	0.87	2.48	3.118 (3)	131
C15—H15B···O2^i^	0.97	2.37	3.120 (3)	134
C15—H15A···Cg1^ii^	0.97	2.75	3.530 (2)	238

Symmetry codes: (i) *x*, −*y*+1/2, *z*−1/2; (ii) −*x*, −*y*, −*z*+2.
